# Structure and Function of A41, a Vaccinia Virus Chemokine Binding Protein

**DOI:** 10.1371/journal.ppat.0040005

**Published:** 2008-01-18

**Authors:** Mohammad W Bahar, Julia C Kenyon, Mike M Putz, Nicola G. A Abrescia, James E Pease, Emma L Wise, David I Stuart, Geoffrey L Smith, Jonathan M Grimes

**Affiliations:** 1 The Division of Structural Biology and The Oxford Protein Production Facility, Wellcome Trust Centre for Human Genetics, University of Oxford, Oxford, United Kingdom; 2 Department of Virology, Faculty of Medicine, Imperial College London, St. Mary's Campus, London, United Kingdom; 3 Leukocyte Biology Section, NHLI Division, Faculty of Medicine, Sir Alexander Fleming Building, Imperial College London, South Kensington Campus, London, United Kingdom; St. Louis University, United States of America

## Abstract

The vaccinia virus (VACV) *A41L* gene encodes a secreted 30 kDa glycoprotein that is nonessential for virus replication but affects the host response to infection. The A41 protein shares sequence similarity with another VACV protein that binds CC chemokines (called vCKBP, or viral CC chemokine inhibitor, vCCI), and strains of VACV lacking the *A41L* gene induced stronger CD8^+^ T-cell responses than control viruses expressing A41. Using surface plasmon resonance, we screened 39 human and murine chemokines and identified CCL21, CCL25, CCL26 and CCL28 as A41 ligands, with K_d_s of between 8 nM and 118 nM. Nonetheless, A41 was ineffective at inhibiting chemotaxis induced by these chemokines, indicating it did not block the interaction of these chemokines with their receptors. However the interaction of A41 and chemokines was inhibited in a dose-dependent manner by heparin, suggesting that A41 and heparin bind to overlapping sites on these chemokines. To better understand the mechanism of action of A41 its crystal structure was solved to 1.9 Å resolution. The protein has a globular β sandwich structure similar to that of the poxvirus vCCI family of proteins, but there are notable structural differences, particularly in surface loops and electrostatic charge distribution. Structural modelling suggests that the binding paradigm as defined for the vCCI–chemokine interaction is likely to be conserved between A41 and its chemokine partners. Additionally, sequence analysis of chemokines binding to A41 identified a signature for A41 binding. The biological and structural data suggest that A41 functions by forming moderately strong (nM) interactions with certain chemokines, sufficient to interfere with chemokine-glycosaminoglycan interactions at the cell surface (μM–nM) and thereby to destroy the chemokine concentration gradient, but not strong enough to disrupt the (pM) chemokine–chemokine receptor interactions.

## Author Summary

As part of the innate immune response (for example to virus infection), the body produces proteins called chemokines, which act by directing white blood cells (leukocytes) to the areas of infection and inflammation. Viruses have evolved mechanisms to fight this immune response. Indeed, so important is this need to protect themselves from the immune system that some viruses, such as poxviruses, devote up to half their genetic information to this battle. We have studied a protein called A41, one component of the response of vaccinia virus (the vaccine used to eradicate smallpox) to the immune system and shown that it interferes with the function of a group of chemokines. These chemokines function by forming concentration gradients along which the white blood cells migrate, and A41 sequesters the chemokines, thereby preventing formation of the gradient. Interestingly, we show also that A41 is very similar in structure to another group of proteins, called vCCIs, that bind chemokines more tightly, blocking their attachment to white blood cells, suggesting that both mechanisms are important for virus virulence.

## Introduction

Chemokines (chemotactic cytokines) comprise a large family of small (∼ 7–14 kDa), secreted proteins that direct the migration of leukocytes into areas of infection and inflammation, as part of the innate immune response [1,2]. They function by binding to cell surface glycosaminoglycans (GAGs) and establishing concentration gradients, which are detected by their cognate seven-transmembrane G-protein coupled receptors (GPCRs) on the surface of immune cells. This leads to activation of leukocytes and macrophages and their consequent chemotaxis to sites of inflammation and infection. Chemokines are classified into four subfamilies called C, CC, CXC and CX3C based on the arrangement of conserved disulphide forming cysteine residues at the N terminus [3]. Despite differences in primary sequence and varied functions within the superfamily, chemokines adopt very similar tertiary structures, with an extended N-terminal loop region followed by a three β stranded sheet arranged in a Greek key motif [4,5], and form strong pM interactions with their cognate receptors.

Poxviruses are a family of large, double-stranded DNA viruses that replicate in the cell cytoplasm [6]. Vaccinia virus (VACV), the prototype member of this family, was the live vaccine used to eradicate smallpox, caused by variola virus (VARV) [7]. The VACV strain Copenhagen genome contains approximately 200 genes [8], although the number of genes varies slightly between strains [9]. About half of these genes are dispensable for virus replication but affect virus virulence, host range or interactions with the immune system. One poxvirus immune evasion strategy is to target the chemokine/chemokine receptor system by encoding viral mimics of chemokines or chemokine receptors, or to express secreted chemokine binding proteins [10,11]. Several poxviruses encode a secreted CC chemokine inhibitor (vCCI) (also previously called vCKBP/T1/35 kDa) that is unrelated to cellular chemokine receptors and binds tightly to CC chemokines with pM affinities [12–14]. vCCI functions by competing for and preventing the interaction of chemokines with their receptors on leukocytes and so blocking their role in the inflammatory response. In contrast, it was proposed that another poxvirus chemokine binding protein, called M-T7 from myxoma virus (MYXV), blocks the binding of chemokines to GAGs and thereby prevents the interaction of the chemokines with the endothelial cell surface [15]. Consequently, a chemokine concentration gradient is not established near the site of infection and leukocytes are not recruited.

The vCCI proteins from leporipoxviruses and orthopoxviruses share several conserved features and therefore might share similar binding modes with chemokines. A collection of structures of vCCI proteins from cowpox virus (CPXV) [16], rabbitpox virus (RPXV) [17] and ectromelia virus (ECTV) [18] reveal a distinct β sandwich topology with no obvious relationship to host chemokine receptors, which are seven-transmembrane GPCRs. One face of the vCCI β sandwich (sheet I) is rather flat and electrostatically bland, whereas the second face (sheet II) is elaborated by a conserved loop, that contributes to the highly negatively charged solvent accessible surface. The acidic surface residues of sheet II are highly conserved in vCCI proteins and were hypothesized to be the chemokine-binding surface for these proteins [16]. A subsequent structure of the complex between RPXV vCCI and human CCL4 confirmed this [17], and structure-based mutational analysis of the ECTV vCCI protein highlighted residues involved in high-affinity interactions between vCCIs and their CC chemokine partners [18]. These recent structures have contributed to an emerging paradigm for vCCI–chemokine interactions, allowing comparisons to be made of the binding modes of these soluble viral proteins and host chemokine receptors.

This paper focuses on another immunomodulatory protein that shares sequence similarity to vCCI and is encoded by VACV gene *A41L*. This gene has also been called 166 using a nomenclature in which genes are numbered sequentially from left to right of the VACV strain Western Reserve (WR) [9]. The A41 protein from VACV strain WR is a secreted 30 kDa glycoprotein and very similar proteins are expressed by camelpox virus, CPXV and all 16 strains of VACV tested [19]. In addition, genome sequencing showed that all orthopoxvirus species and strains studied (over 70 in total) encode a highly conserved *A41L* gene [9]. VACV A41 blocks the recruitment of cells to the site of VACV infection in a rabbit intradermal model, and decreases immunopathology and viral clearance in a mouse intranasal model [19]. When gene *A41L* was deleted from modified VACV Ankara (MVA), an attenuated VACV strain, the resultant virus induced a stronger CD8^+^ T-cell response and conferred better protection against subsequent challenge with a pathogenic strain of VACV [20]. Although A41 has biological properties consistent with those of chemokine binding proteins and is related to a known chemokine binding protein, hitherto, the ligands for A41 were unknown.

A41 has limited sequence similarity to the poxvirus vCCI family of proteins (∼19% sequence identity and ∼40% conservation to both CPXV and ECTV vCCIs) and in addition shares a similar hydropathy profile and has conserved cysteine residues [19]. A41 also shares amino acid similarity with orf virus GIF protein, another member of the vCCI protein family, which binds granulocyte macrophage colony stimulating factor (GM-CSF) and interleukin (IL)-2 [21]. Although many poxviruses encode proteins related to VACV vCCI, there are no close relatives outside poxviruses and so the origin of this gene family is uncertain. VACV and orf virus genomes have quite different G+C content (33% for VACV and 64% for orf virus) and the G+C contents of the VACV gene *A41L* and the orf virus gene encoding the GIF protein match that of the parent virus. Therefore, these genes have been present in these genomes for a long time, or, less likely, were acquired recently from an unknown source with similar G+C content.

To better understand the function of VACV A41 we have identified several chemokines as binding partners and solved the A41 crystal structure and refined it to a Bragg spacing of 1.9 Å. To identify ligands for A41, we expressed and purified it from a VACV-expression system [22] in mammalian cells and screened a panel of chemokines for binding by surface plasmon resonance. A41 bound to CCL25, CCL26, CCL28 and CCL21 with a K_d_ between 10^−7^ to 10^−9^ M. A41 did not inhibit chemokine-induced chemotaxis, although the interaction between A41 and chemokines was inhibited by GAGs, suggesting A41 functions by blocking the binding of chemokines to GAGs. The A41 structure reveals the β sandwich fold observed in the vCCI family of proteins. However, there are notable differences between the structure of A41 and the vCCIs of CPXV [16], RPXV [17] and ECTV [18], notably in the conserved surface loop and the surface charge distribution. In light of the biochemical and structural data we propose a model for the biological function of A41 and the structural interactions that underpin it.

## Results

A41 is secreted from infected cells and has a primary amino acid sequence related to the vCCI family of poxvirus proteins that bind chemokines. However, the ligand(s) for A41 is (are) unknown. To investigate the mechanism of action of A41, we searched for ligands using A41 expressed from mammalian cells using the VOTE expression system from VACV [22]. A recombinant virus (VOTE-A41) was constructed in which the *A41L* gene was driven from an IPTG-inducible bacteriophage T7 RNA polymerase promoter (see Materials and Methods). Cells were infected with VOTE-A41 and expression of A41 was induced by addition with IPTG. A41 was collected from the supernatant of infected cells and purified by ion exchange and size exclusion chromatography (SEC) (Materials and Methods). The final preparation was analysed by SDS-PAGE and contained a single protein of 30 kDa that reacted with an anti-A41 polyclonal antibody [19] by immunoblotting (data not shown).

### A41 Binds to a Subset of CC Chemokines

A41 was coupled to the surface of a CM5 sensor chip and 39 murine and human chemokines (murine chemokines CCL1, CCL2, CCL4, CCL6, CCL7, CCL8, CCL9/10, CCL11, CCL12, CCL19, CCL20, CCL21, CCL22, CCL24, CCL27, CCL28, CXCL2, CXCL5, CXCL9, CXCL10, CXCL11, CXCL12a, CXCL12b, CXCL13, XCL1, CX3CL1, and human chemokines CCL14, CCL15, CCL16, CCL17, CCL18, CCL23, CCL25, CCL26, CXCL3, CXCL4, CXCL6 and CXCL7) were passed sequentially across the chip surface and binding was analysed by surface plasmon resonance (BIACORE). Where possible, mouse chemokines were used initially because VACV strains WR and MVA lacking the *A41L* gene showed a distinct biological phenotype, compared to control viruses, in a mouse model of infection. A control surface coated with ovalbumin (45 kDa, pI 4.5), vCCI (as a positive control for CC chemokines) and a blank surface were analysed in parallel channels on the same chip. The sensorgram for the blank surface was subtracted from that for each protein. Both the chemokines and the immobilised proteins differed in mass and so the binding responses recorded at the end of the injection (RU_eq_) were converted into %R_max_ (where R_max_ is the calculated binding capacity). Table 1 shows the binding activity of immobilised A41 and ovalbumin for the different chemokines expressed as %R_max_. Binding of all chemokines to ovalbumin and the majority of chemokines to A41 were very low (<10% R_max_), but binding to A41 of > 20% R_max_ was observed for hCCL26, hCCL25, mCCL28, hCXCL4, mCCL21 and mCXCL13 (Table 1), suggesting that these might be ligands for A41. vCCI also bound all the CC chemokines bound by A41.

**Table 1:**
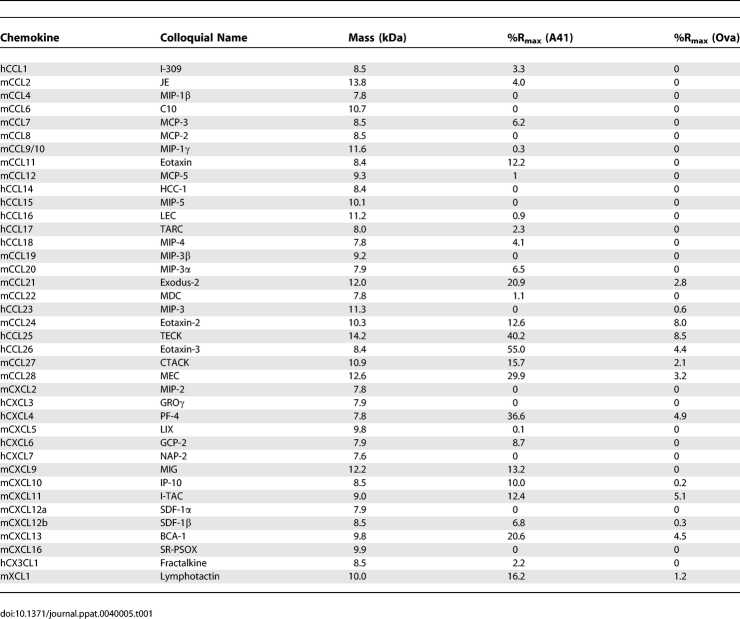
Chemokine Binding (%R_max_) to A41 and Ova Detected by Surface Plasmon Resonance

To investigate these interactions further, kinetic analyses were performed for mouse and human versions of these chemokines using A41 produced from mammalian cells and from E. coli. Binding affinities (K_d_) to A41_VOTE_ and A41*_E. coli_* (mean values from at least two experiments performed on two different chips) are listed in Table 2. K_d_ values of mouse and human CCL25, CCL28, CCL26 and CCL21 are in the range of 8–118 nM, whereas K_d_ values for hCXCL4, hCXCL13 and mXCL1 were higher than 1 μM. Although there were some differences, the binding affinities for each chemokine were generally similar for A41_VOTE_ and A41*_E. coli_*, indicating that A41 can bind to these ligands whether or not it is glycosylated. Overall the K_d_ values of A41 for these human and mouse chemokines are one to three orders of magnitude higher (lower affinity) than the K_d_ values of vCCI for many CC chemokines [13].

**Table 2:**
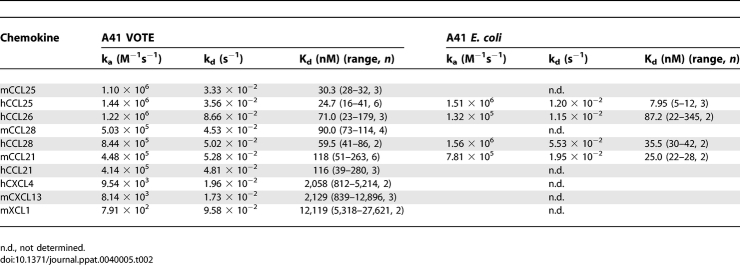
Kinetic Parameters and Affinities of Mouse and Human Chemokines for A41

Poxvirus chemokine binding proteins have been reported to interact with chemokines in two distinct ways. The vCCI protein encoded by VACV and CPXV binds to chemokines such that receptor binding is blocked [13,14,17,23,24] and, the vCCI-chemokine interaction was not inhibited by high concentrations of GAGs, such as heparin [13]. In contrast, the binding of MYXV M-T7 protein to chemokines was blocked by endothelial cell associated GAGs [15]. On this basis a model of the chemokine binding was proposed in which the chemokine receptor binding site and the GAG binding site were considered distinct. The binding of vCCI to CC chemokines would block receptor binding, whereas the inhibition of GAG-chemokine interaction by M-T7 would prevent the establishment of a chemokine concentration gradient around sites of inflammation and the consequent, recruitment of leukocytes. Blocking the binding of a chemokine to its receptor will inhibit leukocyte chemotaxis *in vitro*, whereas blocking binding of a chemokine to GAGs will not. This distinction was exploited to investigate the mechanism of interaction of A41 with chemokines.

### A41 Does Not Inhibit Chemokine-Induced Leukocyte Chemotaxis

**Figure 1:**
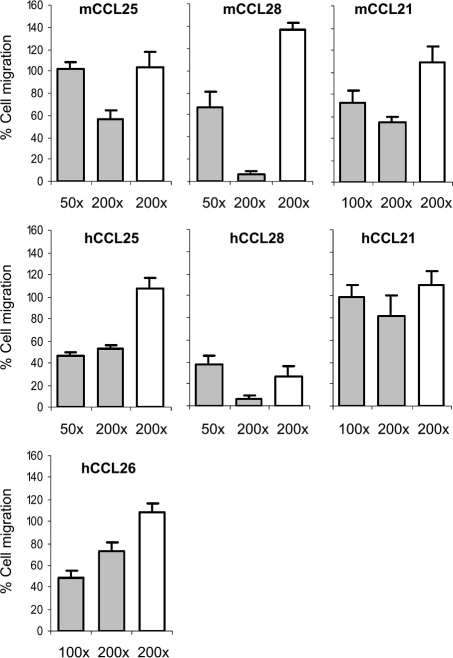
A41 Does Not Inhibit Leukocyte Chemotaxis Induced by Human or Murine CCL21, CCL25, CCL26 and CCL28 A chemokine was incubated with A41 (grey bars) or ovalbumin (open bars) at the indicated molar excess in the bottom of a well of a 96-well chemotaxis plate and 2 × 10^5^ cells were placed in the upper chamber. The plate was incubated for 5 h and the number of cells that had migrated through the membrane was determined. Data are expressed as a percentage of the number of cells that migrated in response to chemokine alone, following subtraction of migration in buffer only, and the error bars represent the S.E.M. based on five or more samples. The number of cells migrating in the absence of chemokine was below 3%.

The ability of A41 to inhibit chemotaxis induced by CCL21, CCL25, CCL26 and CCL28 was examined. Murine L1.2 cells expressing endogenous CCR7 were used to examine human and mouse CCL21-induced chemotaxis; 4DE4 cells expressing CCR3 stably were used to examine human CCL26-induced chemotaxis; and murine L1.2 cells were transfected with plasmids encoding wild type or HA-tagged human CCR9 and CCR10 to examine human and mouse CCL25- and CCL28-induced chemotaxis [25]. In pilot experiments, cells transfected with the HA-tagged CCR9 and CCR10 migrated in response to CCL25 and CCL28 at similar levels to cells transfected with the wild type receptors (data not shown). Thereafter, the HA-tagged versions were used so that cell-surface receptor expression could be verified by FACS using an anti-HA antibody before chemotaxis experiments (data not shown). The concentration of each chemokine that induced optimal chemotaxis of the appropriate cell type was determined and this concentration was used to determine whether A41 can block chemotaxis. A41_VOTE_, A41*_E. coli_* or ovalbumin were incubated at various molar ratios (up to 200:1) with each chemokine and the number of cells that migrated through a membrane in response to this mixture was determined (Figure 1).

No inhibition of chemotaxis was observed with murine and human CCL21 (Figure 1). Similarly, with murine and human CCL25 and hCCL26, even a 200-fold excess of A41 was unable to inhibit chemotaxis by 50% compared to chemokine alone (Figure 1). Only with human and murine CCL28 was A41 able to reduce chemotaxis to ∼10% of levels induced by chemokine alone, but in the latter case chemotaxis was also inhibited ∼65% by a similar molar excess of ovalbumin. Moreover, a 50-fold excess of mCCL28 inhibited chemotaxis by only ∼35% (Figure 1) and this is inconsistent with efficient blockade of chemokine–chemokine receptor interaction. Collectively, these data indicate that A41 is unable to block chemokine-induced leukocyte chemotaxis effectively. These results are consistent with the weaker binding of A41 to chemokines, compared to the binding of those chemokines to their cellular receptors, and contrast with results for VACV vCCI that inhibited chemotaxis efficiently in a dose-dependent manner and at much lower molar concentrations [13].

### A41 Binding Blocks the Chemokine GAG-Binding Domain

**Figure 2:**
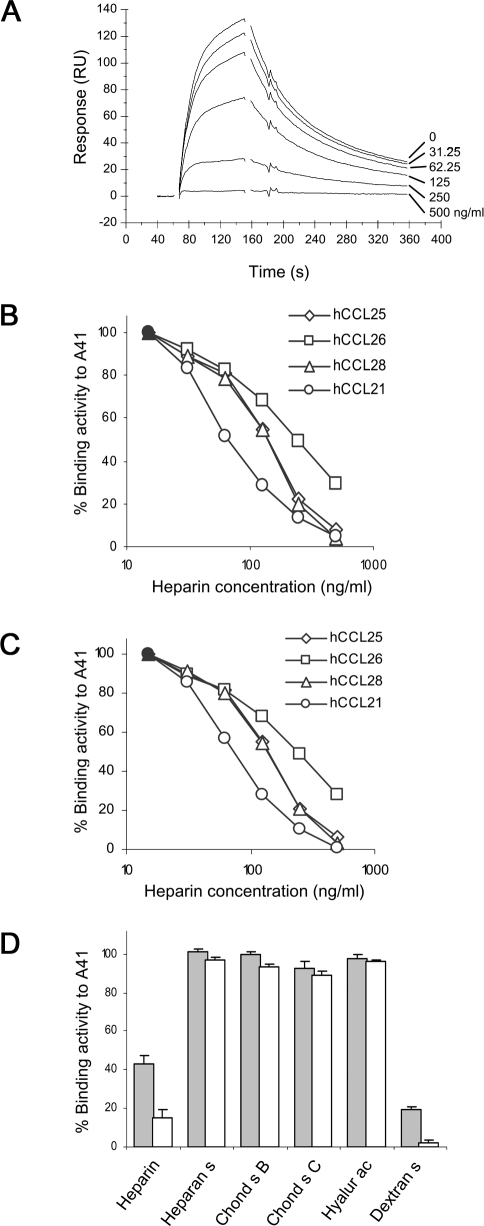
Inhibition of Binding of Chemokines to A41 in the Presence of Heparin Sodium Salt (A) A41_VOTE_ was immobilised on a sensor chip and increasing concentrations (0, 31.25, 62.5, 125, 250, 500 ng/ml) of heparin sodium salt (low molecular weight, MW 4,000–6,000, Sigma) were mixed with hCCL28 and passed across the sensor chip. Chemokine binding to A41 is shown by sensorgrams expressed as RU. (B,C). Heparin inhibits binding of human CCL21, CCL25, CCL26 and CCL28 to A41_VOTE_ (B) and A41*_E. coli_* (C) (filled symbols depict activity with chemokine only). (D) Binding of human CCL25 to A41_VOTE_ in the presence of various sulphated GAGs (heparin, heparan sulphate [heparan s], chondroitin sulphate B [chond s B, = dermatan], chondroitin sulphate C [chond s C], hyaluronic acid [hyalur ac] and dextran sulphate [dextran s]) at 125 ng/ml (grey bars) and 250 ng/ml (white bars). Data are expressed as the percentage binding compared to CCL25 alone.

The failure of A41 to inhibit chemokine-induced chemotaxis and the K_d_ values for its interaction with chemokines (Table 2), suggest that A41 might function by blocking the interaction of chemokines with GAGs. This was investigated by immobilising A41 on a sensor chip, passing hCCL28 across the chip alone or in the presence of increasing concentrations of heparin (sodium salt, MW 4,000–6,000) and measuring hCCL28 binding by BIACORE. The interaction of A41 and hCCL28 was inhibited in a dose-dependent manner and complete inhibition was achieved with 500 ng/ml heparin (Figure 2A). Similarly, heparin inhibited the binding of hCCL21, hCCL25, hCCL26 and hCCL28 to immobilised A41 in a dose-dependent manner when A41 was produced in either mammalian cells (Figure 2B) or E. coli (Figure 2C). The concentration of heparin used to achieve 50% inhibition was ∼100 ng/ml (∼20 nM) and this was lower than used by other investigators to achieve disruption of the M-T7 chemokine binding protein from MYXV with RANTES [15] or the interaction of chemokines with the endothelial cell surface [26]. Apart from heparin, other sulphated GAGs were also tested for their ability to inhibit binding of chemokines to A41. Heparin and dextran sulphate inhibited the hCCL25-A41 interaction, but heparan sulphate, chondroitin sulphate B, chondroitin sulphate C and hyaluronic acid did not (Figure 2D). Similar results were obtained with A41 produced from E. coli or mammalian cells and with hCCL21, hCCL26 and hCCL28 (data not shown). Notably each GAG able to inhibit the hCCL25-A41 interaction was more highly charged than those that did not inhibit.

Collectively these data show that A41 binds a subset of CC chemokines (CCL21, CCL25, CCL26 and CCL28) via a site that overlaps their GAG-binding site, but A41 does not inhibit leukocyte chemotaxis.

### The Structure of A41

**Figure 3:**
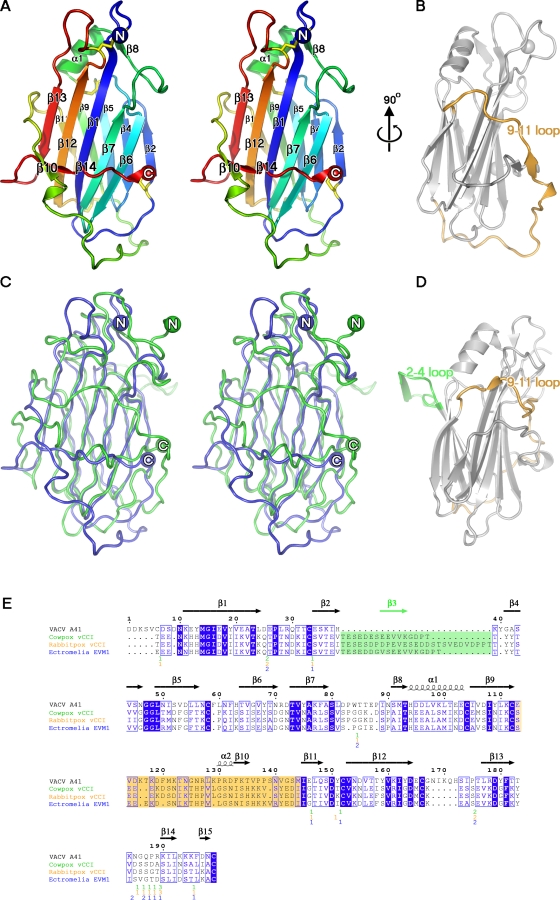
The Structure of VACV Protein A41 (A) Cartoon representation of A41 in stereo view, coloured from blue at the N terminus to red at the C terminus, with the four disulphide bonds shown as yellow sticks. (B) View at 90° to (A), showing the 9–11 loop in yellow. (C) Superposition of A41 and CPXV vCCI in stereo view, with A41 coloured blue and CPXV vCCI coloured green. (D) CPXV vCCI in the same orientation as (B), with the 2–4 loop coloured green and the 9–11 loop coloured yellow. (E) Structure-based sequence alignment between VACV A41 and the vCCIs of CPXV, RPXV and ECTV. Structurally equivalent residues defined by SHP [28] were used to generate a clustalw alignment using shp2clustalw (unpublished program). Identical residues are boxed in blue, and conserved residues are boxed in white. The secondary structure of A41 is shown above the alignment. The 2–4 loop insertion in the vCCIs, which is absent in A41, is highlighted in green, and the 9–11 loop in yellow. With the exception of these loops, residues for the vCCIs that are not matched by A41 are omitted and the position and number of residues removed is indicated under the alignment. This figure was generated with ESPript [64]. PDB IDs 1CQ3, 2FFK and 2GRK were used for the CPXV vCCI, RPXV vCCI, and ECTV EVM1 proteins, respectively. All molecular representations were generated in PyMOL [65].

To understand the structural basis of the action of A41, its crystal structure was determined using protein expressed in E. coli and refolded from inclusion bodies (Materials and Methods). Phase determination was accomplished by MAD analysis of Seleno-methione (SeMet)-labelled crystals. Electron density was observed for residues 26–219 (numbering for mature protein begins at one) and the structure was refined to 1.9 Å (final R = 20.4 and Rfree = 25.2, Table 3). A41 is a single domain protein with the distinctive β sandwich fold seen in the vCCI class of poxvirus chemokine binding proteins. The two β sheets that form the β sandwich, lie parallel to each other (Figure 3A), and are linked by an array of large loops. Five anti-parallel β strands (6, 7, 1, 12 and 13) form β sheet I and define the core of the structure (the naming of secondary structure is as defined in Carfi et al, 1999). The second β sheet (sheet II) is also composed of 5 β strands; 2, 4, 5 and 9 are anti-parallel whilst 11 is adjacent to and parallel with 9. Sheet I is largely buried from solvent by two long enveloping loops on one side and sheet II on the other (the other face of sheet II is exposed to solvent). One of these loops (the 9–11 loop, residues 113–144) wraps around the molecule and connects β strands 9 and 11 (Figure 3B), whilst the second comprises the C terminus and packs tightly against the face of sheet I, and bears strands 14 and 15. Two short β strands (10 and 14) from these loops clip together in front of sheet I. There are also two short helical segments, the first of which (α1) packs against the back of sheet II and is preceded by strand 7. The second, α2, helix comprises a single turn prior to strand 10. Eight cysteine residues in A41 form four disulphide bridges (C6-C166, C33-C199, C58-C104, and C112-C152).

**Table 3:**
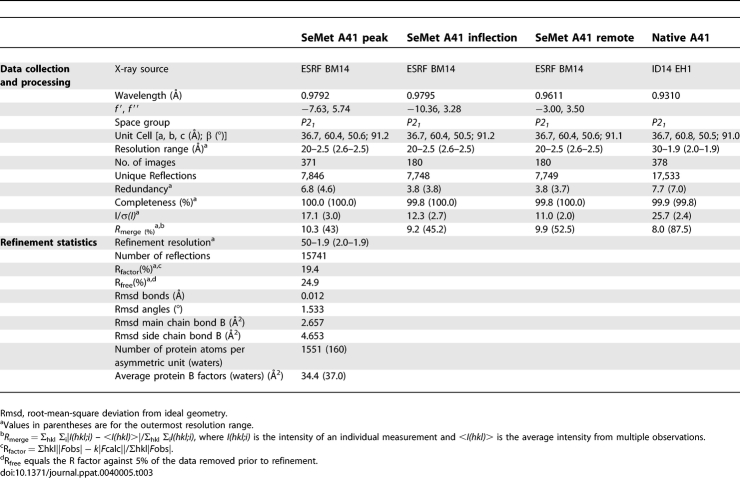
Data Collection and Refinement Statistics for A41

### Structural Comparisons between A41 and Poxvirus vCCIs

Despite sharing only 19% sequence identity with CPXV and ECTV vCCI, the structure of A41 is strikingly similar to the poxvirus vCCI family of secreted chemokine binding proteins. Superposition of A41 with the CPXV vCCI [16] (Figure 3C) aligns 159 Cα atoms (out of 199) with an rmsd of 2.4 Å. The level of similarity is comparable for the recent structures of RPXV [17] and ECTV [18] vCCI proteins (2.4 Å and 2.5 Å rmsds for 159 and 160 residues respectively). In addition, there is notable structural similarity with the M3 protein of murine γherpesvirus68 (γHV68) [27], which binds all four classes of chemokines. However M3 is larger, consisting of N- and C-terminal domains, each possessing β sandwich cores similar to those of the vCCIs. The N- and C-terminal domains can be superposed on A41 with an rmsd of 2.5 Å and 2.8 Å respectively (86 and 47 residues equivalenced).

The most significant structural deviations between A41 and the vCCIs occur in certain surface loops. The first of these, the 9–11 loop (A41 residues 113–144) wraps around the bottom of the β sandwich before arching up the face of sheet I (Figure 3B). The equivalent loop in the CPXV vCCI (residues 133–161, as defined by the structural alignment) has a markedly different orientation and wraps over the top half of sheet I (Figure 3D).

The second major deviation between A41 and the vCCI proteins lies in the orientation of loops projecting from the face of sheet II (Figure 3D). The CPXV vCCI protein [16] contains an extended and highly acidic loop (the 2–4 loop) between strands 2 and 4 that protrudes from the plane of sheet II and makes contact with a symmetry related molecule, giving rise to a crystallographic dimer. This feature is conserved in the RPXV [17] and ECTV [18] vCCI proteins, but is absent in A41 (Figure 3B, 3E) and makes a significant contribution to the charge characteristics of sheet II (see below).

### The Surface Properties of A41 and Modelling the A41-Chemokine Complex

**Figure 4:**
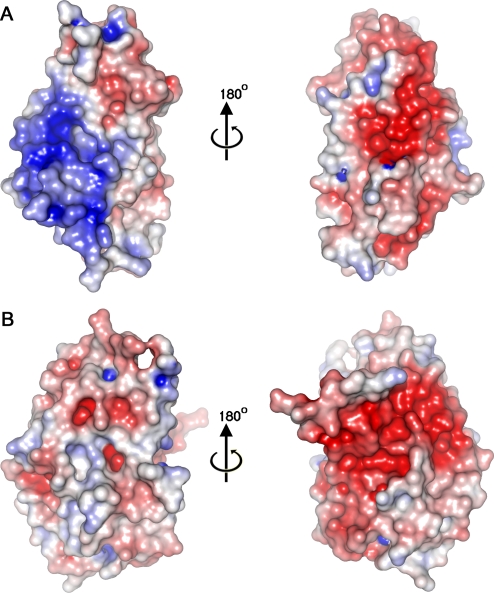
Electrostatic Potential Surfaces of (A) A41 and (B) vCCI The left hand panels show the molecules as viewed in Figure 5A and 5C. The right hand panels show the molecules rotated by 180°. Surface charge was calculated using APBS [66]. Positive charge is coloured blue and negative charge is coloured red.

**Figure 5:**
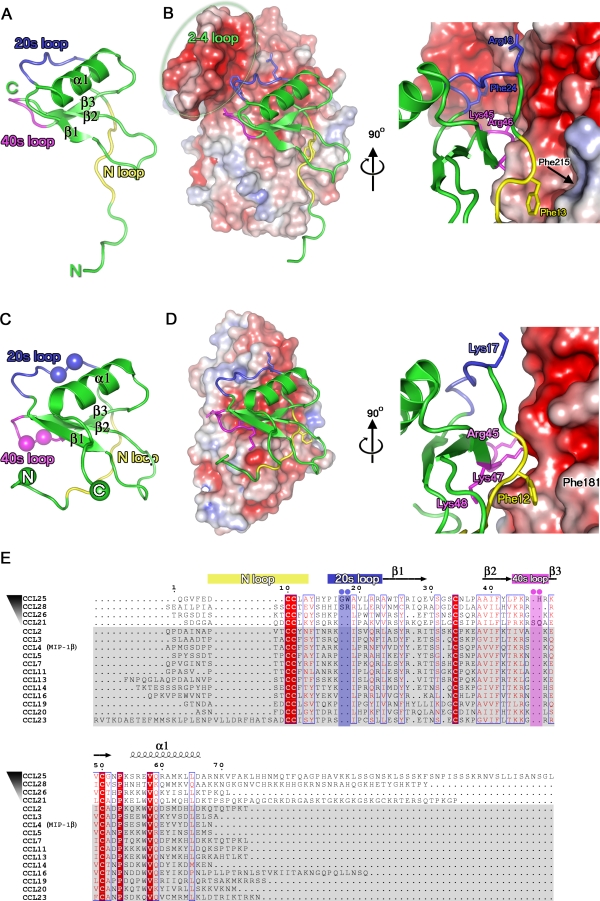
Analysis of Human Chemokines Binding to A41 and Modelling of the A41:CCL26 Complex The structure of CCL4 (A) coloured green, and the N-terminal loop, 20s loop, and 40s loop are coloured yellow, blue and magenta, respectively. (B) The NMR complex of CCL4 with the RPXV: vCCI is shown with CCL4 depicted as in (A) and RPXV vCCI is shown with its electrostatic surface charge. The position of the 2–4 loop is labelled and highlighted by a grey oval. The views are orientated facing the vCCI β sheet II (180° to the view in Figure 3A). (C) The structure of CCL26 coloured as (A). The positions of insertions present in the 20s and 40′s loops of the CC chemokines that bind to A41 are shown with spheres coloured blue and magenta, respectively. (D) A model of the interaction between A41 and CCL26 generated using SHP [28], in an equivalent representation and view to (B). (E) Sequence alignment of CC chemokines that bind to A41 and to vCCIs, created using CLUSTALW [67]. CC chemokines that bind to A41 are ordered in descending order of affinity to A41*_E. coli_*. Those CC chemokines that do not bind to A41, but bind to vCCIs are shaded grey. The secondary structure of CCL26 is shown above the alignment. Sequence numbering is according to the CCL26 sequence.

**Figure 6:**
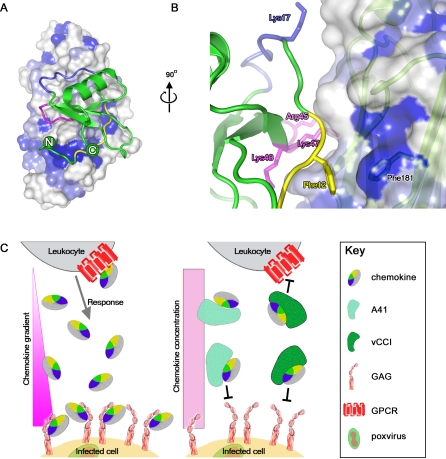
Model of the Interaction of A41 with CCL26, and Schematic Representation of the Biological Mechanism (A) The model of A41 interaction with CCL26 is shown orientated as in Figure 5D, with the structure based sequence conservation between A41 and vCCIs mapped onto the A41 surface (coloured as in Figure 3E, with dark blue patches representing strict sequence identity, the colour scheme for CCL26 is as for Figure 5A). (B) A close up of the interaction between the N-terminal loop of CCL26 and the Phe181 of A41. The A41 surface is shown semitransparent with a cartoon representation of A41 in pale green beneath the surface. (C) Model for mode of action of vCCI-like proteins and A41. The epitope for chemokine receptor on the chemokine is shown in yellow whilst the portion of the chemokine surface that interacts with GAG is coloured blue.

The surface charge properties of A41 are broadly similar to those seen in other poxvirus vCCI proteins, although sheet I exhibits a large patch of positive charge (Figure 4A) whereas in CPXV vCCI sheet I is comparatively uncharged (Figure 4B). On the opposite face of A41 (sheet II) the dominant electrostatic feature is a negatively charged patch, and although this is not conserved in sequence between A41 and the vCCIs this region is negatively charged in the vCCIs and is conserved within that family (E46, D49, E125 and Y62 in particular, CPXV numbering). The complex of RPXV vCCI with chemokine CCL4 demonstrated that this negatively charged surface forms crucial electrostatic interactions with the positively charged 20s and 40s loop of the chemokine (Figure 5A, 5B). This charged surface includes the acidic 2–4 loop that harbours residues E46 and D49 and protrudes from sheet II to lock the chemokine in place. This loop differs in length in the vCCIs (it is 16 and 27 aa long in CPXV and RPXV respectively) and is absent in A41. Mapping structure based sequence alignment between A41 and the vCCIs onto the surface of A41 reveals that below this unconserved charged patch is a region of conservation (Figure 6A, 6B), central to which is a strictly conserved phenylalanine (F181 in A41) which forms a hydrophobic depression on the edge of the β sandwich in A41 and the vCCIs (Figure 5D).

To try and understand this pattern of sequence conservation, we modelled the binding of A41 to its chemokine binding partners using the structure of RPXV vCCI bound to CCL4 (pdb code 2ff3). Although only simple rigid body superpositions were performed to separately position A41 and chemokine hCCL26 (pdb code 1g2s) onto the vCCI-CCL4 complex (maximizing the structural overlap with the appropriate component, program SHP [28]), the quality of the fit of docking is surprisingly good, with few serious steric clashes (11 residues of CCL26 are within 4 Å of 14 residues of A41, Figure 5C, 5D). This A41-CCL26 model reveals that for A41 both the region of sequence conservation, in particular the hydrophobic patch around F181, and the negatively charged patch make close contacts to the chemokine.

Analysis of the RPXV vCCI-CCL4 complex shows that two regions of the chemokine are important for binding, both of which show amino acid sequence conservation. The first region is the N-terminal loop of CCL4, in particular a highly conserved phenylalanine at position 13 in CCL4, which makes contact with the conserved vCCI residues (homologous to F181 in A41) at the edge of the β sandwich that forms the shallow hydrophobic depression. Secondly, positively charged residues in the 20s and 40s loops of CCL4 (particularly positions corresponding to 17, 23, 45, 47 and 48 CCL26, Figure 5E) interact with the negatively charged surface formed primarily by the 2–4 loop of the vCCI. The molecular determinants shaping this binding mechanism are highly conserved in all CC chemokines that bind vCCIs with high affinity [29].

Although the structure of A41 is largely similar to the vCCIs, only a subset of CC chemokines bind to A41 with measurable affinity, and even the tightest interaction is nearly two orders of magnitude weaker than achieved for by the VACV vCCI and CCL3. Nevertheless, the critical residues that define the binding mode of chemokines to vCCIs are conserved in the subset of chemokines (CCL21, 25, 26 and 28) that bind to A41 (Figure 5E). The absence of the 2–4 loop in A41 may explain the specificity of A41 for certain CC chemokines and underlies the weakness of the interaction. This loop is likely to be important for the broad specificity of the vCCI proteins, providing a somewhat flexible electrostatic platform with which to sequester chemokines via their positively charged GAG-binding sites. High affinity might then be conferred by hydrophobic interactions of the N-terminal loop. The lack of the 2–4 loop may therefore restrict the selectivity of A41 to only a few chemokines, although the conservation of a negative surface patch on sheet II is sufficient to form significant electrostatic interactions with certain chemokines. Support for this model comes from an analysis of the amino acid sequence of those chemokines that bind A41 most tightly, which reveals that they possess insertions around the 20s and 40s loops (Figure 5E), with longer loops conferring greater affinity for A41 (Table 2).

## Discussion

This paper provides a structural and functional characterisation of the VACV A41 protein. This protein was reported previously to be secreted from VACV-infected cells and to affect virus virulence [19] and the immune response to infection [20]. Although the A41 protein shares amino acid similarity with the family of poxvirus chemokine binding proteins (vCCI), hitherto its ligand(s) and mechanism of action were unknown. Here we demonstrate that A41 binds a subset of CC chemokines (CCL21, 25, 26 and 28) but the affinity of A41 for these chemokines is 1–3 orders of magnitude lower than the affinity of VACV protein vCCI for a wide range of CC chemokines. Consequently, A41 cannot disrupt the high affinity interactions of chemokines with their cellular receptors and so is unable to inhibit leukocyte chemotaxis in response to these chemokines. However, the affinity of A41 for these CC chemokines is still high enough to predict that A41 will block the interaction of chemokines with GAGs, and consistent with this, high concentrations of GAGs such as heparin and dextran sulphate disrupted the A41-chemokine interaction. Direct attempts to block the interaction of hCCL28 and heparin, immobilised on the surface of BIACORE chips, with excess A41 achieved a 40% inhibition at 250 nM (data not shown). These experiments are complicated by the large number of binding sites present on heparin (mol mass 15,000 Daltons). These observations suggest that A41 functions by blocking the interaction of CC chemokines with GAGs on the endothelial cell surface and thereby disrupting the establishment of a chemokine concentration gradient around the site of infection. So, in the presence of A41, CC chemokines can still bind to their receptors on leukocytes and activate these cells, but the leukocytes would not home to the site of infection. This model (Figure 6C) fits well with the observed increased infiltration of leukocytes into dermal tissue infected with a VACV strain engineered to lack the *A41L* gene [19] and is similar to the proposed mode of action of the MYXV M-T7 protein [15]. It also fits well with observations made with mutant chemokines, such as CCL5, that are deficient in GAG binding. Despite binding and activating chemokine receptors *in vitro*, these chemokines were unable to recruit cells *in vivo*, highlighting the importance of GAG binding to form haptotactic gradients for cells to follow [30].

The structure of the A41 protein was solved to 1.9 Å resolution and shows considerable similarity to the family of chemokine binding proteins (vCCI) from poxviruses, even though these proteins share only ∼19% sequence identity with A41. Like the CPXV, ECTV and RPXV vCCI proteins [16–18], A41 is composed of a β sandwich comprising two parallel β sheets connected and partially covered by extended loops. Four disulphide bonds contribute to the protein stability. The extended loops of A41 differ significantly from other vCCI family proteins and these changes are likely to affect the affinity and specificity of the chemokines bound. A notable difference between A41 and other vCCI proteins is the absence of the 2–4 loop (acidic β strand 3, numbered according to [16]), which in the RPXV vCCI-CCL4 complex helps to lock the chemokine in place via electrostatic interactions [17]. The absence of this loop in A41 may explain its reduced affinity for chemokines. However, this apart, the basic interaction of vCCI-CCL4 and the modelled A41-CCL26 interaction is remarkably similar. As for vCCI-CCL4, the predicted binding of A41 with CCL26 is aided by the packing of conserved hydrophobic residues in the N-terminal loop of the chemokine in a hydrophobic depression on the A41 surface. This depression is formed primarily by F181, which is strictly conserved in A41 and in vCCI proteins from all sequenced orthopoxviruses. The selectivity of A41 for CCL21, 25, 26 and 28 is likely to be influenced by the lack of the 2–4 loop in A41 and by the insertion of residues in the 20s and 40s loops of these chemokines (Figure 5E).

The nature of the chemokines bound by A41 also provides an explanation for the increased immunogenicity of VACV strains engineered to lack the *A41L* gene, and in particular the induction of enhanced levels of antigen specific CD8^+^ T cells in the secondary lymphoid organs [20]. CCL21 is a pivotal molecule for priming T-cell responses, co-stimulating the expansion of naïve CD4^+^ and CD8^+^ T cells and inducing Th1 polarization [31]. It recruits CCR7^+^ T cells and DCs into the lymph nodes [32] and is responsible for the movement of CD4^+^ T cells within the lymph node [33,34]. CCL21 is up-regulated during a febrile response, promoting uptake of lymphocytes into the lymph nodes across the high endothelial venules [35]. This involvement in the formation and maintenance of a specific anti-viral immune response means that CCL21 is a logical target of many viral proteins. Notably, simian and human immunodeficiency viruses [36], hepatitis C virus [37] and murine γ-herpesvirus 68 [38] have evolved different ways to block its activity.

CCL25 is also involved in the formation of a T-cell response, although its expression is mainly localised in the thymus and small intestine. It is responsible for the homing of CCR9-expressing T-cell progenitors to, and their migration through, the thymus [39,40]. It is also important for the development of immune responses in the gut mucosa. CCL28 is also expressed by the mucosal epithelia of the gut, where it attracts CD4^+^ and CD8^+^ resting T cells [41] and has broad antimicrobial properties [42]. An impairment of its effector functions by A41 would be expected to affect the T-cell response, but probably in the mucosa rather than the lymphoid organs.

It is interesting that VACV encodes two soluble proteins that bind CC chemokines (vCCI and A41). Of these, A41 is the more conserved and is expressed by every VACV strain tested [19] and indeed every sequenced orthopoxvirus species and strain [9], now more than 75 viruses. In contrast, vCCI is expressed by only nine out of 15 VACV strains tested [13]. However, each protein can influence virus virulence: deletion of gene *A41L* from VACV strain WR, which does not express vCCI [13], caused enhanced cellular infiltration and reduced virulence in rabbit skin [19]; conversely, insertion of the *vCCI* gene from VACV strain Lister into VACV strain WR, which expresses A41, attenuated the virus in a murine intranasal model, characterized by reduced mortality and weight loss, decreased virus replication and spread, and a reduced recruitment of inflammatory cells into the lungs [43]. These proteins therefore have distinct roles, consistent with their different binding specificities, affinities and modes of action.

In the light of the results for A41 we can reformulate the model whereby vCCI-like molecules block the chemokine receptor epitope, whilst A41 or MT-7-like molecules block the GAG binding epitope of the chemokines. In our revised model (Figure 6C) the binding sites on chemokines for GAGs and chemokine receptors largely overlap and the functional distinction between the two classes of molecules arises simply from the differences in binding affinity. vCCI binds sufficiently tightly to block the interaction of chemokines with their receptors and GAG binding is also blocked. In contrast, A41 acts by blocking the establishment of a chemokine concentration gradient by competing with GAG binding only. Having both strategies may be advantageous for a virus because some chemokines, such as CCL21, can exert some of their functions without forming concentration gradients (a process known as chemokinesis) [44]. This ability, coupled with the anti-viral potency of CCL21, may mean that some viruses have evolved more than one way to target this chemokine and inhibit its various functions.

## Materials and Methods

### Expression of A41 in the VOTE system.

The VACV strain Western Reserve (WR) *A41L* gene was amplified from plasmid pA41 [19] by PCR using primers GGGGATTAATATGTACTCGTTAGTATTTG and GGGGGAATTCTTAACAATTATCAAATTTTTTC. The DNA was digested with AseI and EcoRI and ligated into plasmid pVOTE2 [22] that had been digested with NdeI and EcoRI. The sequence of the *A41L* open reading frame within the resultant plasmid pVOTE2-A41 was verified by DNA sequencing. Virus VOTE-A41 was constructed by transfection of pVOTE2-A41 into RK_13_ cells infected with virus vT7lacOI [22], and selection of recombinant virus using methodology described previously [22].

### Purification of A41 from mammalian cells.

To express A41 in mammalian cells, monolayers of RK_13_ cells were infected with VOTE-A41 at 5 p.f.u. / cell for 90 min, washed and then incubated in culture medium containing 3 mM IPTG and 190 mM NaCl. At 24 h p.i. A41 was purified from the culture medium. The medium was concentrated ∼50-fold using centrifugal concentrators with a 10 kDa cut-off (Amicon). Virus particles were inactivated by treatment with 20 μg/mL psoralen and long-wave UV for 20 min on ice [45] and removed by ultracentrifugation (80 min, 35,000 x *g*). The supernatant was then applied to a 20 ml Resource Q column (GE Healthcare) pre-equilibrated with 2 column volumes of TE (50 mM Tris-HCl pH 7.0, 1 mM EDTA) containing 50 mM NaCl. Unbound proteins were washed out with two column volumes of low salt (50 mM NaCl) TE buffer, and bound proteins were then eluted with a salt gradient (50 – 400 mM NaCl). The protein content of each fraction was analysed by SDS-PAGE, Coomassie blue staining and immunoblotting with anti-A41 antibody [19]. Fractions containing the A41 protein peak were pooled and concentrated to 200 μl using a 2 ml centricon (Amicon). The concentrated samples were then purified further by SEC on a Superdex S75 HR 10/30 column in PBS. Fractions containing A41 were pooled and concentrated to 200 μl using a 2 ml centricon (Amicon). SDS-PAGE showed a single 30 kDa protein that reacted with the anti-A41 antibody by immunoblotting.

### Surface plasmon resonance (BIACORE).

The carboxymethylated dextran surface of a CM5 sensor chip (GE Healthcare) was activated with 50 mM N-hydroxysuccinimide (NHS) and 200 mM N-ethyl-N′-(3-dimethylaminopropyl)-carbodiimide hydrochloride (EDC). Proteins A41 and ovalbumin in 10 mM sodium acetate pH 4 were then coupled to different flowcells on the chip at a rate of 5 μL/min using the BIACORE Application Wizard to a target of 1000 response units (RU) for the binding experiments. The chip surface was then deactivated and unbound protein was removed by injecting a pulse of 1 M ethanolamine hydrochloride pH 8.5 across all flowcells. To perform screening for analyte binding, the murine chemokines CCL1, CCL2, CCL4, CCL6, CCL7, CCL8, CCL9/10, CCL11, CCL12, CCL19, CCL20, CCL21, CCL22, CCL24, CCL27, CCL28, CXCL2, CXCL5, CXCL9, CXCL10, CXCL11, CXCL12a, CXCL12b, CXCL13, XCL1, CX3CL1, and the human chemokines CCL14, CCL15, CCL16, CCL17, CCL18, CCL23, CCL25, CCL26, CXCL3, CXCL4, CXCL6 and CXCL7 (Peprotech Ec) were passed over the chip surface sequentially, at 50 nM (25 μL/min, 2 min injection) in HBS-EP buffer (10 mM Hepes pH 7.4, 150 mM NaCl, 3 mM EDTA, 0.005% surfactant P20). The surface of the chip was regenerated in between each analyte with 10 mM glycine pH 3. The value observed from the empty flowcell (Fc1) was deducted electronically from the other flowcells. For kinetic analyses of A41 binding, a target of 250 RU of purified A41_VOTE_ and A41*_E.coli_* was coupled to the surface of a new CM5 sensor chip. Various concentrations (3.125 nM – 50 nM) of each chemokine in HBS-EP were passed over the chip (50 μl/min, 2 min injection). To calculate the dissociation constant (K_d_) for each analyte, data were analysed using a simultaneous k_a_/k_d_ fitting procedure and a Langmuir (1:1) model. The quality of the fit was assessed using the residuals plot and the χ^2^ value.

To investigate if GAGs inhibited the binding of A41 and chemokines, a new chip was prepared by immobilising 1000 RU of purified A41_VOTE_ and A41*_E.coli_* as described above for the binding assays. Chemokines were incubated at room temperature for 1 h in the presence of increasing concentrations (0, 31.25, 62.5, 125, 250, 500 ng/ml) of heparin sodium salt (low molecular weight, MW 4,000–6,000, Sigma-Aldrich) or various other sulphated GAGs (125 or 250 ng/ml heparan sulphate sodium salt, chondroitin sulphate B sodium salt (=dermatan), chondroitin 6-sulphate sodium salt (=chondroitin sulphate C), hyaluronic acid and dextran sulphate, all Sigma-Aldrich) and BIACORE analysis performed as above.

### Chemotaxis assays.

Assays were performed as described previously [46]. Briefly, different concentrations of A41 or Ova were incubated with mouse (m) or human (h) CCL21, CCL25 or CCL28 or human CCL26, in 31 μl in the bottom well of 96-well ChemoTx^TM^ plates (Receptor Technologies, Oxford, UK). Five to 8 samples were analysed for each concentration. A 5 μm filter was placed onto the wells and 2 × 10^5^ target cells in 20 μl were placed onto the filter above each well. Plates were incubated for 5 h at 37 °C and cells that had migrated through the membrane were counted with a haemocytometer. Target cells were mouse L1.2 cells that express CCR7 naturally (used for mCCL21 and hCCL21) [47], 4DE4 cells expressing CCR3 (used for CCL26) [25] and L1.2 cells transfected transiently with plasmids expressing wild type or N-terminally HA-tagged human CCR9 and CCR10 (http://www.cdna.org/) as described [48]. The surface expression of HA was verified before each experiment by FACS analysis, as described [49], using a monoclonal anti-HA antibody (Covance) at 1/100 dilution, and FITC-conjugated goat anti-mouse at 1/20 dilution (Dako) and the appropriate isotype control (Sigma-Aldrich). Initial experiments were performed using wild type and HA tagged alleles to ensure that the HA tag did not interfere with receptor function. To determine the concentration of each chemokine that induced the optimal chemotaxis, chemokine concentrations between 5 and 150 nM were tested. Concentrations used were 10 nM for hCCL21 and mCCL21, 20 nM for mCCL25, 50 nM for hCCL25, 40 nM for CCL26, 100 nM for mCCL28 and 60 nM for hCCL28.

### Cloning and expression of Escherichia coli A41.

The *A41L* gene (encoding residues 21–219, excluding the secretion signal) was amplified by PCR from VACV strain WR DNA with KOD Hot Start DNA polymerase (Novagen) using forward primer 5′-ggggacaagtttgtacaaaaaagcaggcttcgaaggagatagaaccatggcacatcaccaccaccatcacGATGATAAATCGGTATGCGATTC-3′ and reverse primer 5′-ggggaccactttgtacaagaaagctgggtctcaTTAACAATTATCAAATTTTTTCTTTAATATTTTACG-3′. The forward primer encoded a start codon and an N-terminal His_6_ tag. Both primers featured the *attB* site of the gateway cloning system (Invitrogen) that was used to subclone the purified PCR product into the pDEST14 expression vector. The expression plasmid was shown to be mutation free by sequencing.

Native A41 was expressed in the E. coli strain Rosetta(DE3)pLysS. Cultures were grown at 37 °C for 4 h in GS96 medium (Invitrogen), supplemented with Overnight Express Autoinduction System 1 (Novagen), before being incubated at 25 °C overnight. Selenomethionine (SeMet) labelled protein was expressed in the E. coli methionine auxotrophic strain B834(DE3) (Novagen). Cells were cultured at 37 °C in SeMet medium (Molecular Dimensions Ltd.) supplemented with 40 mg/l SeMet, until the OD_595_ reached 0.6. Cultures were then cooled to 20 °C, A41 expression was induced by addition of 0.5 mM Isopropyl-βD-thiogalactopyranoside and the culture was left to incubate overnight. All cultures were harvested by centrifugation (6000 × g, 4 °C, 20 min) and stored at −80 °C until use.

### Refolding and purification.

Cell pellets were resuspended in phosphate-buffered saline (PBS) with 0.5 % (v/v) Tween-20, and lysis was performed by sonication (Sonics Vibracell) on ice. Inclusion bodies, comprising mainly A41, were isolated by centrifugation (30,000 x *g*, 8 °C, 10 min) and subjected to several washes in triton wash buffer (50 mM Tris-HCl pH 8.0, 100 mM NaCl, and 0.5 % (v/v) triton X-100) with centrifugation (as above) between washes to re-collect inclusion bodies. A final wash was performed in detergent free buffer (50 mM Tris-HCl pH 8.0 and 100 mM NaCl). The inclusion bodies were then dissolved overnight at 4 °C in a denaturing buffer containing 50 mM Tris-HCl pH 8.0, 100 mM NaCl, 6 M guanidine hydrochloride and 10 mM DTT, followed by centrifugation (30,000 × *g*, 8 °C, 30 min) to remove undissolved waste. The supernatants containing denatured A41 were stored at −20 °C before refolding by rapid dilution with stirring of 20 mg of denatured inclusion bodies into 200 ml of refolding buffer containing 200 mM Tris-HCl pH 8.0, 1 M L-Arginine, 6.5 mM cysteamine, 3.7 mM cystamine, plus 1 EDTA-free protease inhibitor cocktail tablet (Roche). Refolding reactions were incubated at 4 °C for between 24–48 h, followed by concentration to a volume of 5 ml in a vivacell 250 concentrator (Vivascience) with a 10 kDa cut-off. Concentrated protein was purified by SEC on a HiLoad 16/60 Superdex 200 column (GE Healthcare), into a final buffer of 20 mM Tris-HCl pH 7.5 and 200 mM NaCl. One hundred percent incorporation of selenomethionine was confirmed by mass spectrometry.

### Crystallization and data collection.

Prior to crystallization, purified A41 was concentrated by ultrafiltration to 3 mg/ml in a 10 kDa cut-off concentrator (Vivascience). For both native and SeMet-substituted A41, initial vapour diffusion crystallization experiments were performed at 21 °C in 300 nl drops (protein/precipitant ratio of 2:1) using a Cartesian robot [50,51]. Crystals were grown from a mother liquor of 0.2 M potassium fluoride, 20 % polyethylene glycol 3350. Based on this result, further optimisations were performed [52]. Crystals of A41 belong to space group P2_1_, (unit cell dimensions a = 36.6Å, b = 60.8Å, c = 50.4Å, β = 91.0°), and contain one molecule per asymmetric unit with an estimated solvent content of 50 %. Both native and SeMet crystals were flash frozen at 100K in mother liquor containing 20 % glycerol. Diffraction data to 1.9 Å resolution were collected for the native crystals at the European Synchrotron Radiation Facility (ESRF), beamline ID14 EH1. A multiple wavelength anomalous diffraction (MAD) analysis was performed at 2.3 Å resolution on a SeMet crystal at beamline BM14 at the ESRF. All oscillation images were processed and reduced using the *HKL* software suite [53] (Table 1).

### Structure solution and refinement.

The structure of A41 was solved using the MAD method. Each monomer of A41 contains four SeMet residues and the positions of these were determined using SHELXD [54] and initial phases computed with SHELXE [55] as part of the HKL2MAP package [56]. Density modification used RESOLVE [57], and the resulting electron density map was of excellent quality, allowing an automatic chain trace to be performed with Arp/wARP [58], which built 160 out of 199 residues. The remainder of the structure was modelled in the program O [59]. Initial refinement was performed using program CNS [60]. The 1.9 Å native data were isomorphous to the SeMet data, permitting refinement to be directly extended to this higher resolution in the program REFMAC [61] with iterative rebuilding in COOT [62]. For the final model the *R*_work_ is 19.4 % and the *R*_free_ is 24.9 % (Table 1). The stereochemical quality of the structure was assessed using the MOLPROBITY program [63]. The structure has good stereochemistry with 94.3 % of residues lying in the most favoured regions of the Ramachandran plot.

### Modelling of the CC26-A41 complex.

The CC26-A41 complex was modelled by superimposing A41 onto the vCCI component of the RPXV vCCI-CCL4 complex (pdb code 2FF3) (159 equivalences with an rmsd of 2.4Å), and then superposing CCL26 (pdb code 1G2S) onto the CCL4 component (59 equivalences with an rmsd of 1.8 Å). All superpositions were done automatically using SHP [28].

## Supporting Information

### Accession Numbers

Coordinates and structure factors have been deposited in the Protein Data Bank (PDB; http://www.rcsb.org/pdb/) with accession numbers 2vga and 2vgasf.
